# P01.56. Does practicing Reiki alter the electromagnetic field of heart and hands of practitioners?

**DOI:** 10.1186/1472-6882-12-S1-P56

**Published:** 2012-06-12

**Authors:** A Baldwin, W Rand, G Schwartz

**Affiliations:** 1 University of Arizona, Tucson, USA; 2 Center for Reiki Research, Michigan, USA

## Purpose

This study attempted to repeat experiments performed 20 years ago that detected exceptionally high strength electromagnetic fields (100 nT) from the hands of several energy healers. The equipment utilized in this replication attempt was far more sensitive, and the magnetic shielding used to block confounding fields from electrical lines far more effective, than in the original studies.

## Methods

Using a Magnes 2500 WH SQUID at Scripps Research Institute, San Diego, the electromagnetic field from the hands and heart of each of 3 Reiki masters was measured for 5 minutes when they were: (i) not practicing Reiki; (ii) sending Reiki to a distant person; and (iii) sending Reiki to a person in the room. Similar measurements were made on 4 Reiki-naïve volunteers before and after they received Reiki attunements enabling them to run Reiki energy through themselves.

## Results

For all subjects, under all conditions, sensors closest to the heart and the hands produced spikes of 2pT corresponding to the heartbeat. Recordings from two Masters and one volunteer showed a low intensity sine wave oscillation of 0.25-0.3Hz (intensity 0.1-0.5pT) whether or not they were practicing Reiki. This oscillation probably reflected respiratory sinus arrhythmia, as judged by comparison with recent previous studies. These signals were not detected in the original studies. No segments of electromagnetic field intensity greater than 3pT were seen in any recording in the current study.

## Conclusion

These results suggest that practicing Reiki does not routinely produce high intensity electromagnetic fields from the palms. The previous findings could have been from a few exceptional healers. Alternatively, it is possible that energy healing is stimulated by attunement to an external environmental radiation, such as the Schumann resonance, which was blocked in the present study by the strong magnetic shielding in the room containing the SQUID.

